# Curcumin as therapeutics for the treatment of head and neck squamous cell carcinoma by activating SIRT1

**DOI:** 10.1038/srep13429

**Published:** 2015-08-24

**Authors:** An Hu, Jing-Juan Huang, Rui-Lin Li, Zhao-Yang Lu, Jun-Li Duan, Wei-Hua Xu, Xiao-Ping Chen, Jing-Ping Fan

**Affiliations:** 1 Department of Otolaryngology, Gongli Hospital, Second Military Medical University, Pudong New Area, Miaopu Road 219, Shanghai, 200135, China; 2 Department of Cardiology, Shanghai Chest Hospital, Shanghai Jiaotong University, Huaihai Xi Road 241, Xuhui District, Shanghai 200030, China; 3 Department of Gerontology, Xinhua Hospital, Shanghai Jiaotong University School of Medicine, Kongjiang Road 1665, Shanghai, 200092, China; 4 Department of Otolaryngology-Head & Neck Surgery, ChangZheng Hospital, Second Military Medical University, Fengyang Road 415, Huangpu District, Shanghai, 200003, China

## Abstract

SIRT1 is one of seven mammalian homologs of Sir2 that catalyzes NAD^+^-dependent protein deacetylation. The aim of the present study is to explore the effect of SIRT1 small molecule activator on the anticancer activity and the underlying mechanism. We examined the anticancer activity of a novel oral agent, curcumin, which is the principal active ingredient of the traditional Chinese herb Curcuma Longa. Treatment of FaDu and Cal27 cells with curcumin inhibited growth and induced apoptosis. Mechanistic studies showed that anticancer activity of curcumin is associated with decrease in migration of HNSCC and associated angiogenesis through activating of intrinsic apoptotic pathway (caspase-9) and extrinsic apoptotic pathway (caspase-8). Our data demonstrating that anticancer activity of curcumin is linked to the activation of the ATM/CHK2 pathway and the inhibition of nuclear factor-κB. Finally, increasing SIRT1 through small molecule activator curcumin has shown beneficial effects in xenograft mouse model, indicating that SIRT1 may represent an attractive therapeutic target. Our studies provide the preclinical rationale for novel therapeutics targeting SIRT1 in HNSCC.

Cancer is the second leading cause of age-related mortality in humans. Recent studies have provided evidence for the potential clinical utility of targeting histone deacetylase (HDAC) enzymes in haematological malignancies^1^,^2^,^3^ and other squamous cell carcinoma^4^,^5^,^6^. Sirtuin 1 (SIRT1) is known to play an important role in maintaining metabolic homeostasis in multiple tissues in an NAD^+^-dependent fashion^7^,^8^,^9^,^10^. While many HDACs have been extensively studied, the role of SIRTs in head and neck squamous cell carcinomas (HNSCC) remains undefined.

SIRT1 is one of seven mammalian homologs of Sir2 that catalyzes NAD^+^-dependent protein deacetylation, yielding nicotinamide and O-acetyl-ADP-ribose^11^. SIRTs are distinct from the class I/II/IV HDACs because they do not have any sequence similarity with other HDACs and are not sensitive to HDAC inhibitors^12^,^13^,^14^. To date, seven human sirtuins have been identified. SIRT1 is also known to modify both histones (histone H1, histone H3 and histone H4) and non-histone proteins that are involved in apoptosis, cell growth, metabolism, caloric restriction and cell senescence^8^,^9^,^10^. Furthermore, SIRT1 modulates p53, nuclear factor (NF)-κB, peroxisome proliferator activated receptor-γ coactivator (PGC)-1α, liver X receptor (LXR) and Fork head transcription factors^8^.

There are conflicting data *in vitro* as to whether SIRT1 will be found to act as a tumor suppressor or as an oncogene. On the one hand, recent studies demonstrated that SIRT1 levels are reduced in some other types of cancers, and that SIRT1 deficiency results in genetic instability and tumorigenesis, while overexpression of SIRT1 attenuates cancer formation^15^,^16^,^17^,^18^. On the other hand, SIRT1 has been considered as a tumor promoter because of its increased expression in some types of cancers and its role in inactivating proteins that are involved in tumor suppression and DNA damage repair^19^,^20^,^21^,^22^. SIRT1-deficiency resulted in an increased tumor formation in p53-null mice^18^. Another study showed that SIRT1 inhibits proliferation of pancreatic cancer cells expressing pancreatic adenocarcinoma up-regulated factor^23^. Recently, the data demonstrated that the reduction in tumor development is caused by the ability of SIRT1 to deacetylate β-catenin and promote cytoplasmic localization of the nuclear-localized oncogenic form of β-catenin^17^. These studies support the potential of SIRT1 as tumor suppressor, and provide the rationale for medical research of activators of SIRT1 in the treatment of cancer.

In the present study, we examined the efficacy of one such novel SIRT1 activator curcumin in HNSCC using *in vitro* and *in vivo* models. Curcumin (diferuloylmethane) is a polyphenol derived from the plant Curcuma longa, commonly called turmeric. Extensive research over the last 50 years has indicated this polyphenol can both prevent and treat cancer^24^,^25^. In this study we tested whether curcumin, through increasing SIRT1 activity, could modulate SIRT1 functions *in vitro* and *in vivo* and ultimately impact on the regulation of tumor formation and growth.

## Methods

### Cell culture and proliferation assay

HNSCC cell lines including FaDu and Cal27 cells were cultured with DMEM low-glucose medium supplemented with 10% inactivated fetal bovine serum and 100 μg/ml penicillin and 100 μg/ml streptomycin under standard culture conditions^26^ (37 °C, 95% humidified air and 5% CO2). Supernatant and cell lysates were collected at 3 days after reseeding. A total of 3 × 10^4^ cells per well were seeded onto fibronectin-coated 24-well plates, and proliferation assays were performed according to the manufacturer’s instructions. After pretreatment with 7 μM/10 μM curcumin for 6, 12, 24 and 48 h, or left untreated. Then, cells were re-suspended and counted. Curcumin was obtained from Shanghai Laboratory Animal Co Ltd (SLAC, Shanghai, China). Each condition was assessed in triplicate.

### Deacetylase assay (Fluor de Lys)

Deacetylase activity of SIRT1 was measured using the Fluor de Lys Deacetylase kit (BIOMOL international Inc, USA) as described previously^27^. Briefly, 4 × 10^4^ cells were plated in 50 μl of medium containing flour de lys substrate. The plate was incubated for 5 h at 37 °C, and followed by addition of 100 μl of 2 μmol/l Trichostatin A developing solution according to the manufacturer’s instructions. After further incubation for 15 min at 37 °C, the fluorescence was measured using Multi-Mode Microplate Readers (Sunnyvale, CA, USA).

### Cell apoptosis assay

Cell apoptosis was quantified using the Annexin V/propidium iodide (PI) detection kit (Beyotime, Shanghai, China) and analyzed by flow cytometry. Cells (2 × 10^5^/well) were plated in 6-well dishes and treated with 10 μM curcumin or no curcumin exposed to 10 μM KU55933 or no KU55933. After treatment, collected cells were incubated in 400 μl binding buffer with 5 μl Annexin V-FITC and 5 μl PI in dark for 15 min at room temperature.

### Migration assay (*In vitro*)

Transwell Insert Assays using Transwell migration chambers (BD Biosciences) with 6.5-mm-diameter polycarbonate filters (8 μm pore size) were utilized to measure migration as previously described^26^. In brief, cells were pretreated with curcumin for 1–3 hours as indicated. Thereafter, the bottom chambers were filled with 600 μL of DMEM media containing all supplements. FaDu and Cal27 cells (3 × 10^4^ per well) were seeded in top chambers in 100 μL DMEM media without serum. Cells were allowed to migrate for 8  h, and <5% of cell death was observed with curcumin treatment.

### Capillary-like tubule structure formation assay (*In vitro*)

*In vitro* angiogenesis was assessed by Matrigel capillary-like tubule structure formation assay as described previously^28^. Matrigel-Matrix (BD Biosciences, Franklin Lakes, New Jersey, USA) was pipetted into pre-chilled 96-well plates (50 μL matrigel per well) and polymerized for 45 min at 37 °C. FaDu and Cal27 cells (2 × 10^4^ per well) in complete media were seeded in matrigel coated plates. Then culture plates were exposed to curcumin for 1–3 hours as indicated. Images were acquired under a fluorescent microscope (IX-71; Olympus, Tokyo, Japan) with 12.8 M pixel recording digital color cooled camera (DP72; Olympus). Capillary tubule branch points were counted in six randomly selected fields per well, and used as an index for tubule formation.

### Xenograft tumor model (*In vivo*)

All animal experiments were approved by the Institutional Animal Care and Use Committee of the Shanghai Jiaotong University School of Medicine and group housed four per cage on a 12:12 light: dark cycle with free access to food and water. The methods were carried out in accordance with the approved guidelines. The xenograft tumor model was performed as previously described^29^. Female athymic nude (nu/nu) mice (3–4 weeks old) were purchased from Shanghai Laboratory Animal Co Ltd (SLAC, Shanghai, China). The mice were bred and maintained under specific pathogen-free conditions in the Animal Facility, Shanghai Jiaotong University. FaDu (5 × 10^6^) cells were inoculated subcutaneously into the right rear flank of nude mice. The volume of the implanted tumor was measured at every 2 days with a vernier caliper, using the formula: V = L × W^2^/2; When tumors were measurable approximately 1 weeks after FaDu cells injection, mice were treated orally with vehicle alone (20% PEG400 deionized water) or curcumin (200 mg/kg) for 4 weeks on a seven consecutive days/week schedule. Curcumin was suspended in 0.5% carboxymethylcellulose. Drug was administered orally through gavage in a volume of 2 ml/kg of body weight. KU-55933 was dissolved in PBS to a concentration of 200 μM. Mice treated once daily with KU-55933 (30 mg/kg) subcutaneous administration, once daily. The mice were sacrificed and the tumors were weighd 5 weeks after inoculation.

### Western blotting and protein quantification assay

Western blotting analyses were performed as described previously^30^ using antibodies against caspase-3, caspase-7, caspase-8, caspase-9, poly(ADP) ribose polymerase (PARP) (Cell Signaling, Beverly, MA, USA), p53, VEGF, MMP-2, phosphorylated-ataxia telangiectasia mutated (pATM), phosphorylated-checkpoint kinase-2 (pCHK2), phosphorylated-IκB and GAPDH (Beyotime, Shanghai, China). GAPDH was used as a loading control. Densitometry of protein bands was acquired using an EC gel documentation system (Alpha Innotec, Kasendorf, Germany), and bands were analysed using the spot densitometry analysis tool (Alpha Ease FC software, version, 4.1.0).

### Immunofluorescence antibody assay (IFA)

Cancer tissue samples were processed for hematoxylin-eosin staining (H&E) and immunofluorescence (IF) with activated caspase-3 (AC3) antibodies for the detection of apoptosis. Tissues were also evaluated for the presence of CD31 and existing mature blood vessels by IF staining. The presence of characteristic nuclear foci after immunostaining for Ki-67 was considered to represent the induction of proliferation under microscope. Monolayers were rinsed once in phosphate-buffered saline (PBS), fixed with cold methanol for 30 min and blocked with 5% bovine serum albumin in PBS with Ca^2+^ and Mg^2+^ for 60 min. After further incubation for 15 min at 37 °C, the fluorescence was measured using a fluorescence microscope (Olympus BX-40) and a Leica DFC 300FX camera. Analyses were performed by a single blinded researcher^30^.

### RNA extraction and Quantitative real-Time PCR assay

Total RNA isolation from samples was performed using the TRIzol reagent (Invitrogen, CA, USA) according to the manufacturer’s instructions. The concentrations and quality of the RNA were determined with a NanoDrop ND-1000 spectrophotometer (NanoDrop Technologies, DE, USA) and agarose gel electrophoresis. The expression of VEGF mRNAs was assayed using TaqMan mRNA reverse transcription assays (Applied Biosystems) following the manufacturer’s instructions. The primers for VEGF were using forward (5′-GAGGGCAGAATCATCACGAA-3′) and reverse (5′-GGGAACGCTCCAGGACTTAT-3′); MMP-2 forward (5′-TTGCTGCCACAAGAACTG-3′) and reverse (5′-TTGAAAGACTGGGAGAAG-3′). The reaction conditions indicated in the manufacturer’s manual were used and the reaction mixtures were incubated at 50 °C for 2 min,95 °C for 10 min, followed by 40 cycles of 95 °C for 15 sec and 60 °C for 1 min. The ∆∆Ct method for relative quantization was used to determine mRNA expression levels. The ΔCt value was calculated by subtracting the Ct of GAPDH from the Ct of the mRNA of interest. The ∆∆Ct value was calculated by subtracting the ∆Ct of the reference sample from the ΔCt of each sample. The fold-change was determined as 2^−∆∆Ct^.

### Statistical analysis

Statistical significance of differences observed in drug-treated versus control cultures was determined by the Student’s t test. The distribution of the cell survival data was preliminarily verified using the Kolmogorov-Smirnov test, and the data sets were analyzed by one-way analysis of variance. The minimal level of significance was P < 0.05. Tumor volume in mice was measured using the GraphPad prism (GraphPad Software, SanDiego, CA, USA).

## Results

### Curcumin targets against SIRT1

**Figure 1:**
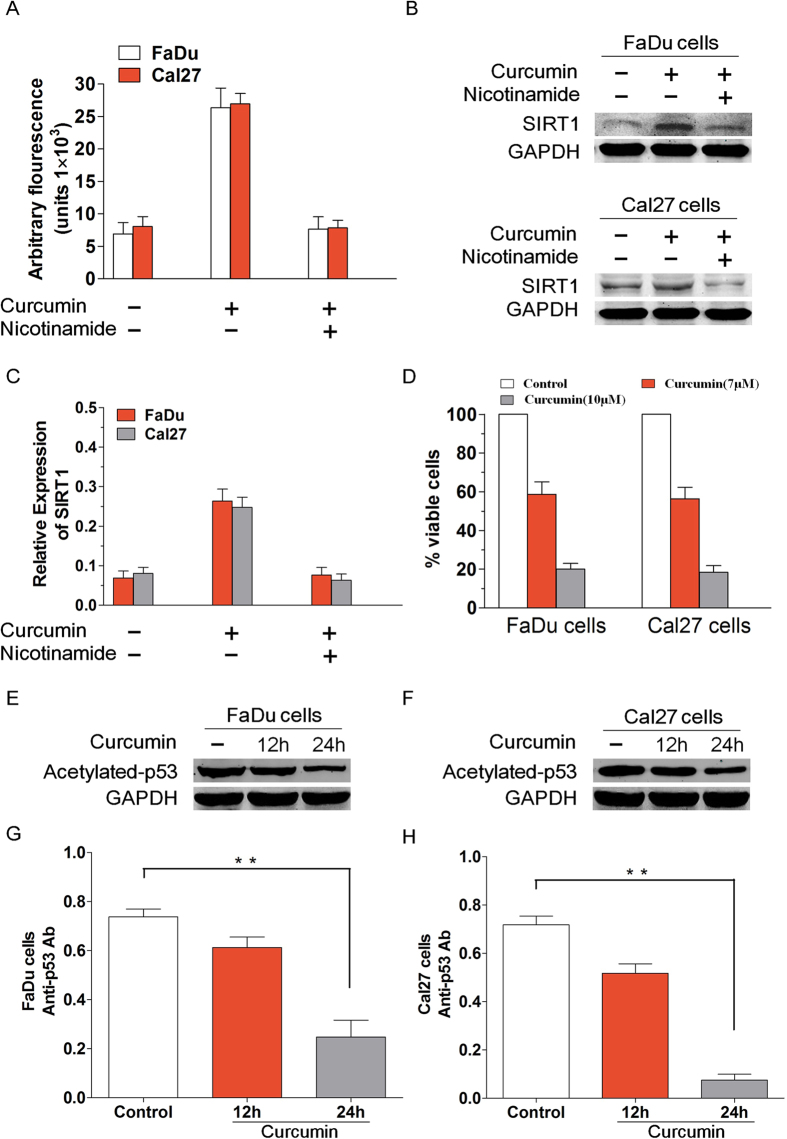
Curcumin induces SIRT1 enzyme activity *in vitro*. (**A**) FaDu and Cal27 cells were treated with curcumin (7 μmol/l) in the presence or absence of SIRT1 inhibitor nicotinamide for 6 h and harvested. Then the extracts were analysed for SIRT1 enzyme activity. Treatment of FaDu and Cal27 cells with curcumin markedly increased the deacetylating activity; conversely, pre-treatment of cells with nicotinamide significantly blocked curcumin-triggered deacetylating activity. (**B–C**) To further confirm curcumin activity on SIRT1, we explored SIRT1 expression by Western blotting in FaDu and Cal27 cells treated or not with curcumin. Treatment of carcinoma cells with curcumin markedly increased the SIRT1 expression. However, pre-treatment of FaDu and Cal27 cells with nicotinamide significantly blocked curcumin-triggered SIRT1 activity. (**D**) We determined a decrease in cell viability using MTT assay and this was due to anti-proliferative activity of curcumin. FaDu as well as Cal27 cells were treated with or without curcumin at the indicated concentrations for 24 h, followed by assessment for cell viability using MTT assays. Raw data from MTT assays were normalized to the % of viable cells in control versus curcumin-treated carcinoma cells. (**E–F**) FaDu and Cal27 cells were treated with curcumin and then the protein lysates were subjected to Western blot analysis with antibodies specifically against acetylated p53 and GAPDH. (**G**) Curcumin pretreatment of FaDu cells diluted acetylated p53 expression level at 12 h, with increasing effect at 24 h. (P < 0.01, n = 3) (**H**) When the effect of curcumin on acetylated p53 expression level of Cal27 cells was tested, a significant reduction was observed at 24 h. (P < 0.01, n = 3) Blots shown are representative of three independent experiments. Data were expressed as mean ± SD. **p < 0.01

For investigated the effect of the curcumin on the modulation of SIRT1 activity, we used different experimental strategies. Firstly, we utilized the Fluor de Lys Deacetylase assay to measure whether curcumin affects SIRT1 deacetylase enzymatic activity. Treatment of FaDu and Cal27 cells with curcumin markedly increased the deacetylating activity; conversely, pre-treatment of cells with nicotinamide, an inhibitor of SIRT1, significantly blocked curcumin-triggered deacetylating activity (Fig. 1A). Secondly, to further confirm curcumin activity on SIRT1, we explored SIRT1 expression by Western blotting in FaDu and Cal27 cells treated or not with curcumin. Treatment of carcinoma cells with curcumin markedly increased the SIRT1 expression. However, pre-treatment of FaDu and Cal27 cells with nicotinamide significantly blocked curcumin-triggered SIRT1 activity (Fig. 1B–C). We determined a decrease in cell viability using MTT assay and this was due to anti-proliferative activity of curcumin. FaDu as well as Cal27 cells were treated with or without curcumin at the indicated concentrations for 24 h, followed by assessment for cell viability using MTT assays. (Fig. 1D). Western blot analysis using antibodies specific against acetylated p53, a known substrate of SIRT1, showed a marked decrease in acetylated state of p53 in curcumin-treated FaDu and Cal27 cells (Fig. 1E–F). Curcumin pretreatment of FaDu cells diluted acetylated p53 expression level at 12 h, with increasing effect at 24 h (Fig. 1G). When the effect of curcumin on acetylated p53 expression level of Cal27 cells was tested, a significant reduction was observed at 24 h (Fig. 1H). Our data is showing that curcumin-induced biological effects occur via SIRT1.

### Curcumin inhibits HNSCC cells growth and proliferation

**Figure 2:**
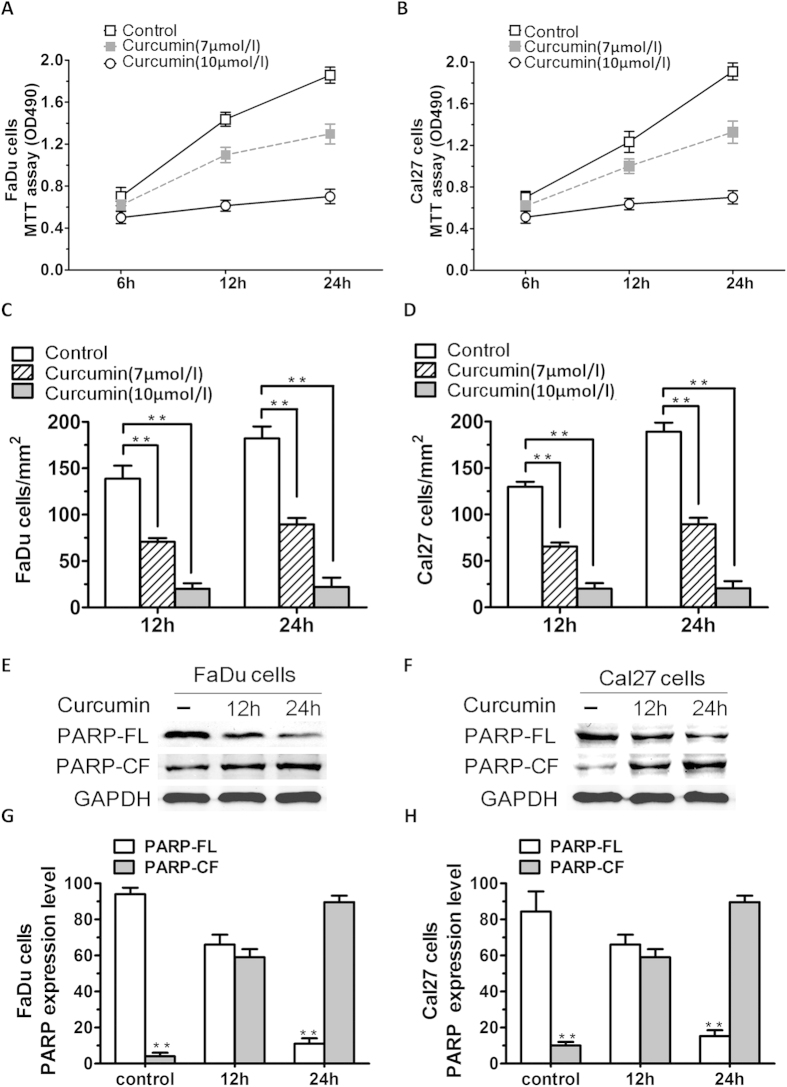
Curcumin inhibits HNSCC cells growth and proliferation *in vitro*. (**A,B**) Cell viability assays measuring growth and proliferation were performed in FaDu and Cal27 cells. (**C,D**) Human HNSCC cell lines (FaDu and Cal27 cells) were treated with various concentrations (7 μmol/l and 10 μmol/l) of curcumin for 24 h. A concentration-dependent decrease in viability of all cell lines was observed in response to treatment with curcumin. (P < 0.01, n = 3) (**E,F**) FaDu and Cal27 cells were treated with curcumin and harvested. Whole cell lysates were subjected to Western Blot analysis with anti-PARP, or anti-GAPDH (FL, full length; CF, cleaved fragment). (**G,H**) Treatment of both FaDu and Cal27 cells with curcumin triggered a marked increasing in proteolytic cleavage of PARP (P < 0.01; n = 3). Data were expressed as mean ± SD. **p < 0.01, versus control group.

To determine the anticancer potential of curcumin and its potential mechanism of action through targeting of SIRT1 *in vitro*, cell viability assays measuring growth and proliferation were performed in FaDu and Cal27 cells. Human HNSCC cell lines (FaDu and Cal27 cells) were treated with various concentrations (7 μmol/l and 10 μmol/l) of curcumin for 24 h, followed by assessment for cell viability using MTT assay. A significant concentration-dependent decrease in viability of all cell lines was noted in response to treatment with curcumin (Fig. 2A,B). 7 μmol/l curcumin inhibited FaDu and Cal27 cells growth at rates about 50% to those of control cells. Potent anti-proliferation effect of 10 μmol/l curcumin compared with no stimulation in FaDu and Cal27 cells (Fig. 2C,D; P < 0.01; n = 3). We next examined whether the curcumin-triggered decrease in viability was due to apoptosis. FaDu and Cal27 cells were treated with curcumin (7 μmol/l) and harvested. Then whole cell lysates were subjected to Western Blot analysis with anti-PARP, or anti-GAPDH (Fig. 2E,F). Moreover, treatment of both FaDu and Cal27 cells with curcumin triggered a marked increase in proteolytic cleavage of PARP, a signature event during apoptosis (Fig. 2G,H; P < 0.01; n = 3).

### Curcumin blocks migration and tubule formation of HNSCC cells *in Vitro*

**Figure 3:**
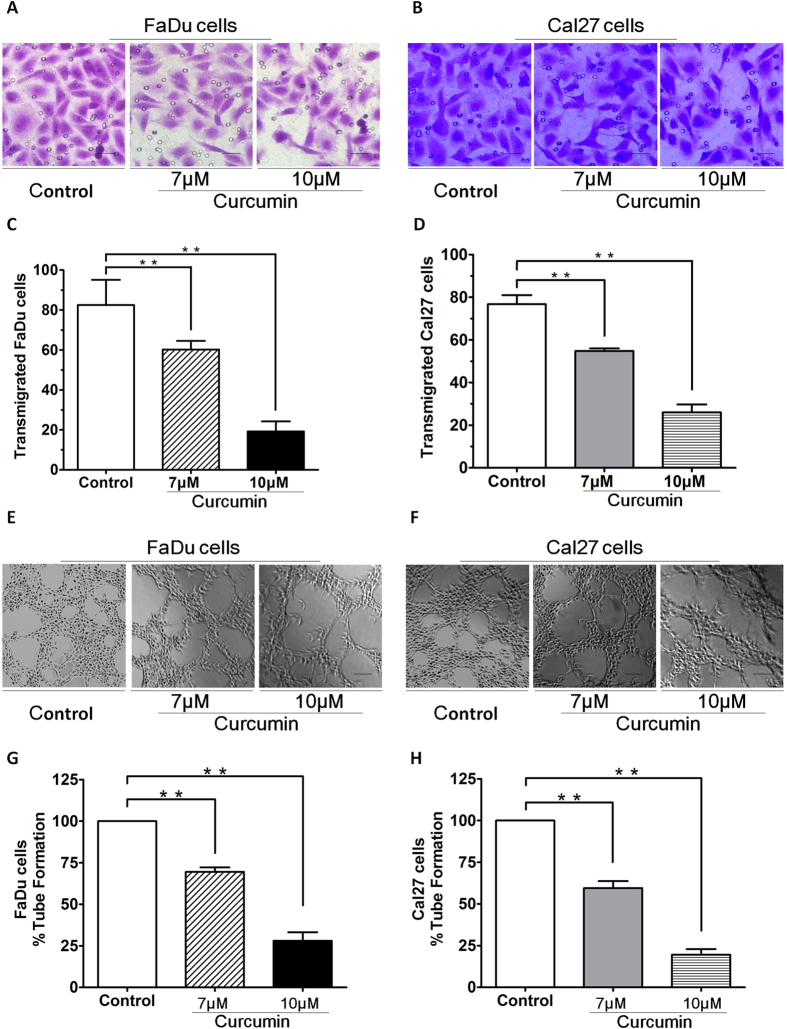
Curcumin inhibited HNSCCs migration. (**A,B**) Curcumin significantly inhibited FaDu and Cal27 cells migration, as evidenced by a decrease in the number of crystal violet-stained cells. (**C,D**) Pretreatment of FaDu and Cal27 cells with curcumin resulted in inhibition of cell transmigration. 10 μM curcumin pretreatment of FaDu and Cal27 cells inhibited transmigration, which was statistically significant difference to the effect of control group. (**E,F**)Vasculogenic mimicry was measured *in vitro* using 3D-Matrigel capillary-like tubule structure formation assays: FaDu and Cal27 cells plated onto Matrigel form capillary-like tubule structures similar to *in vivo* neovascularization. FaDu and Cal27 cells were seeded in 96-well culture plates precoated with Matrigel; and then examined for tubule formation using an inverted microscope. (**G,H**)Tubule formation was markedly decreased in a dose-dependent manner in curcumin-treated cells. Cells remained >95% viable before and after performing the migration assay, excluding the possibility that drug-induced inhibition of migration was due to cell death. Data were expressed as mean ± SD. **p < 0.01.

Many studies have shown that migration and angiogenesis play an important role in the progression of HNSCC. To determine the anti-migration and anti-vasculogenic mimicry potential of curcumin and its potential mechanism of action through induction of SIRT1 activity *in vitro*, transwell insert systems and tubule formation assays measuring transmigration and tubule formation were performed in FaDu and Cal27 cells. Importantly, curcumin significantly inhibited FaDu and Cal27 cells migration, as evidenced by a decrease in the number of crystal violet-stained cells (Fig. 3A,B). Pretreatment of FaDu and Cal27 cells with curcumin resulted in inhibition of cell transmigration. 10 μM curcumin pretreatment of FaDu and Cal27 cells inhibited transmigration, which was statistically significant difference to the effect of control group (Fig. 3C,D). We next utilized *in vitro* capillary-like tubule structure formation assays to assess whether curcumin induces anti-vasculogenic effects. FaDu and Cal27 cells were seeded in 96-well culture plates precoated with Matrigel; and then examined for tubule formation using an inverted microscope (Fig. 3E,F). Tubule formation was markedly decreased in a dose-dependent manner in curcumin-treated cells (Fig. 3G,H). These findings suggest that curcumin blocks migration and Vasculogenic mimicry.

### Mitochondria mediate anticancer activity of curcumin

**Figure 4:**
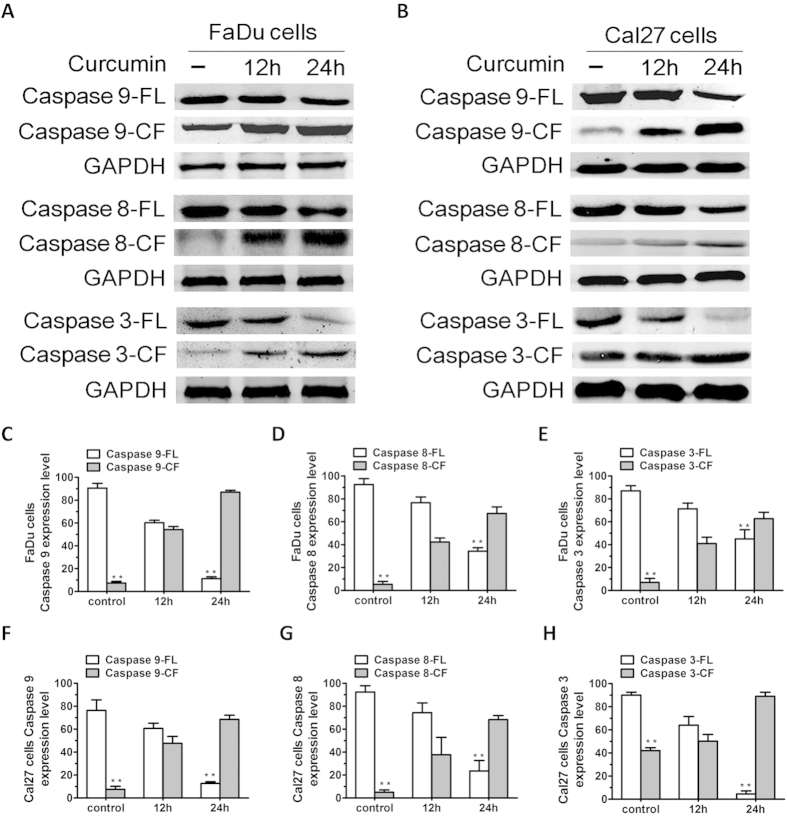
Curcumin triggers activation of both intrinsic (caspase-8) and extrinsic (caspase-9) apoptotic signalling. (**A,B**) FaDu and Cal27 cells were treated with curcumin (7 μM) for the indicated times and harvested. Total proteins were subjected to Western blot analysis (WB) with anti-caspase-9, caspase-8, caspase-3, or GAPDH Abs. (**C–H**) Bar graph represents quantification of the percentage of caspase/ GAPDH. Blots shown are representative of three independent experiments. Results are mean ± SD of three independent experiments (n = 3; P < 0.005).FL, full length; CF, cleaved fragment.

We next assessed whether the SIRT1 activation is due to a direct interaction of the mitochondria. Mitochondria mediate apoptotic signal via activation of the cell death initiator caspase, pro-caspase 9 (intrinsic apoptotic pathway). Therefore, we next assessed potential activation of SIRT1 using native curcumin. Similarly, Fas associated death-domain (FADD) protein is an essential part of the death-inducing signal complexes that assemble upon engagement of tumor necrosis factor receptor family members resulting in proteolytic processing and autoactivation of pro-caspase-8 (extrinsic apoptotic pathway). Our study show that curcumin induced activation of both caspase-8 and caspase-9 apoptotic pathways (Fig. 4). Both caspase-8 and caspase-9 are known to activate a common downstream effector pro-caspase-3, and our data further showed that curcumin triggered caspase-3 activation, as evidenced by caspase-3 cleavage (Fig. 4).

### Curcumin-induced apoptosis is associated with activation of SIRT1 signal pathway

**Figure 5:**
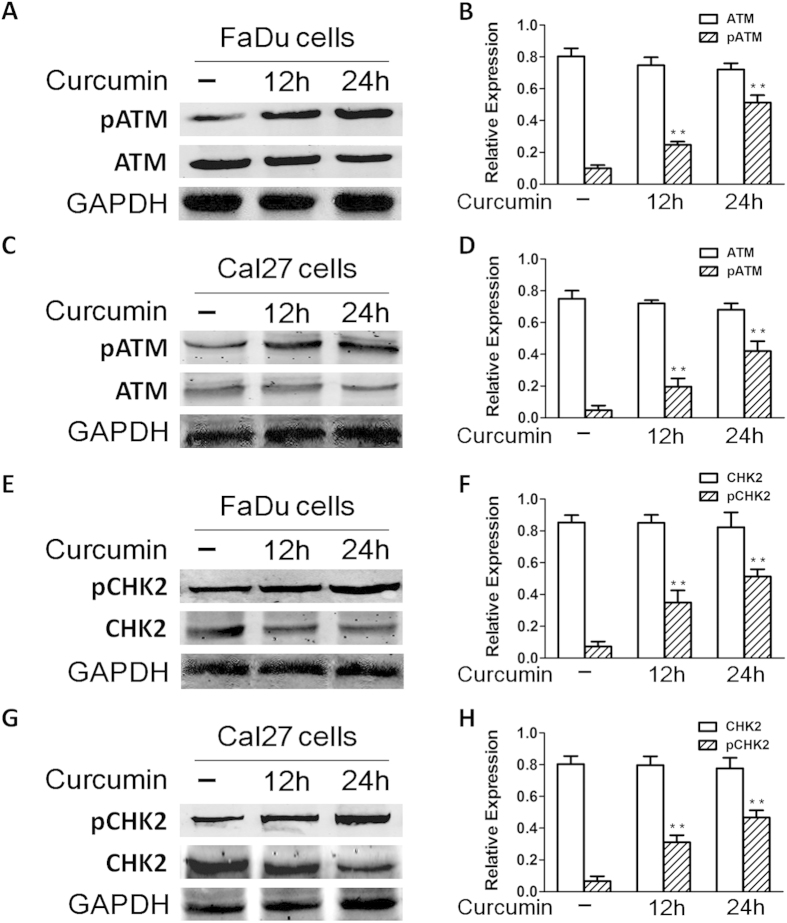
Curcumin-induced apoptosis is associated with activation of ATM/CHK2 signal pathway. (**A–D**) FaDu and Cal27 cells were treated with curcumin (7 μM) and harvested; total proteins were subjected to Western blot analysis with anti-phosphorylated ATM (anti-pATM) or anti-ATM Abs. Blots shown are representative of three independent experiments. (**E–H**) FaDu and Cal27 cells were treated with curcumin (7 μM) and harvested; total proteins were subjected to Western blot analysis with anti-phosphorylated CHK2 (anti-pCHK2) or anti-CHK2 Abs. Blots shown are representative of three independent experiments.

Given that SIRT1 is linked to DNA damage response, and curcumin targets SIRT1, we next examined whether curcumin triggers the ATM mediated apoptotic signal pathway. For more direct visualization of substrates in the assay, we also utilized Western blot analysis using two separate antibodies, one specific for ATM and another specific for CHK2. Treatment of FaDu and Cal27 cells with curcumin induced phosphorylation of ATM (pATM). We further confirmed these data by quantification of pATM using Western blot assay: curcumin triggered a significant increase in pATM levels in FaDu and Cal27 cells (Fig. 5A–D). Examination of CHK2, a known downstream target of ATM, showed a marked increase in phosphorylation in response to curcumin (Fig. 5E–H), suggesting that curcumin-induced FaDu and Cal27 cells apoptosis occurs via activation of the ATM/CHK2 pathway.

**Figure 6:**
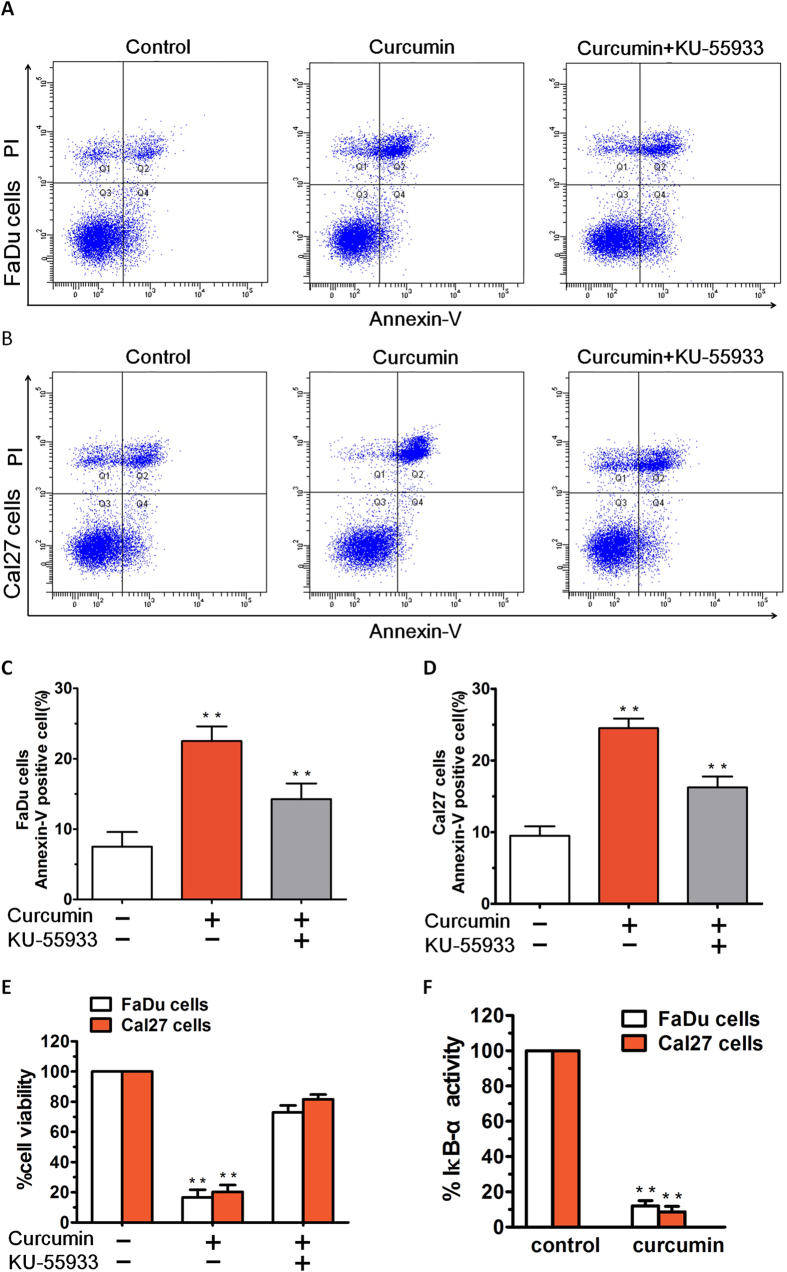
Annexin-V/PI staining showed that curcumin treatment induced apparent early and late apoptosis in HNSCCs. (**A,B**) After treatment with curcumin (7 μM), the FaDu cells and Cal27 cells groups showed early and late apoptosis at rates of 17.20% and 21.60%, respectively. (**C,D**) After treatment with 10 μM KU55933, the apoptosis rate was significantly reduced, not only in Fa**D**u cells group, but also in Cal27 cells group. (**E**) FaDu and Cal27 cells were treated with curcumin in the presence or absence of the ATM inhibitor KU55933 for 24 h, followed by assessment for cell viability using MTT assays. (**F**) FaDu and Cal27 cells were treated with curcumin (7 μM) for 24 h, and extracts were then analysed for NF-κB activity by measuring phosphorylated IκB-α. Data presented are means ± SD (n = 3; P < 0.05).

Annexin-V/PI staining showed that 10 μM curcumin treatment for 36 h induced apparent early and late apoptosis in FaDu and Cal27 cells. As shown in Fig. 6A–B, after treatment with curcumin (7 μM), the FaDu cells and Cal27 cells groups showed early and late apoptosis at rates of 17.20 and 21.60%, respectively. After treatment with 10 μM KU55933, the apoptosis rate was significantly reduced, not only in FaDu cells group, but also in Cal27 cells group (Fig. 6C–D). The results indicate that KU55933 could reduce the apoptosis induced by curcumin in FaDu and Cal27 cells. Importantly, blockade of ATM using a biochemical inhibitor KU55933 partially inhibited curcumin-induced decrease in cell viability (Fig. 6E). Taken together, these data suggest that the anticancer activity of curcumin predominantly relies on the ATM-CHK2/caspase 8/9 pathway.

Besides activation of pro-apoptotic signal cascades, we also examined whether curcumin affects growth and survival signal pathways. In the present study, we demonstrate the specific biologic sequelae of NF-κB activation and blockade on FaDu and Cal27 cells growth and survival. The inhibitor curcumin inhibits phosphorylation of IκB-α in FaDu and Cal27 cells and modestly directly inhibits their growth. In this study, treatment of FaDu and Cal27 cells with curcumin significantly blocked NF-κB activity, evidenced by a decrease in IκB-α activity (Fig. 6F). These findings suggest that the inhibition of NF-κB activity contributes to the anticancer activity of curcumin.

### Curcumin inhibits HNSCC cells growth (*In vivo*)

**Figure 7:**
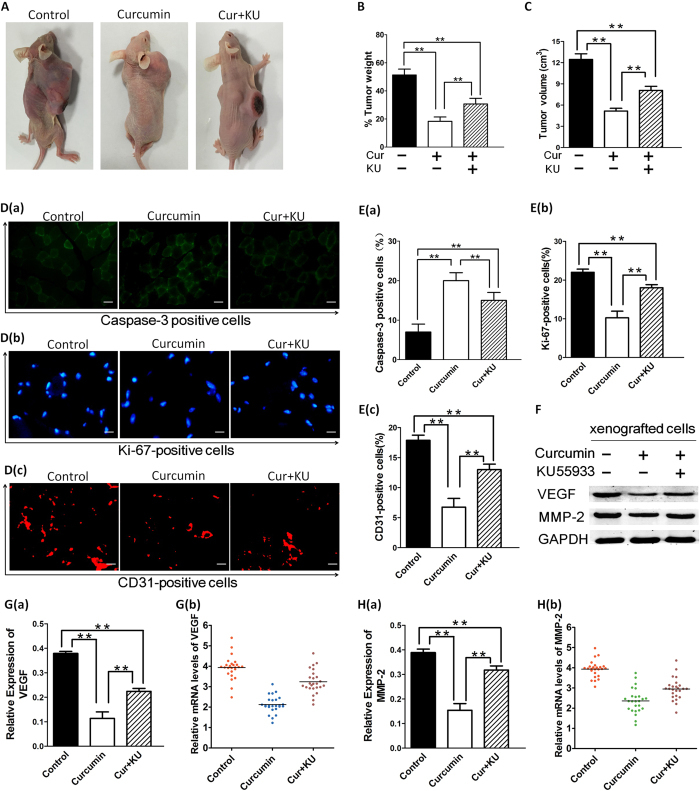
Curcumin inhibits growth of xenografted FaDu cells in mice. (**A–C**) FaDu cells alone (5 × 10^6^ cells/mouse) were implanted in the right rear flank of female null mice (5–7 weeks of age at the time of tumor challenge). On Day 27–30, mice were randomized to treatment groups and treated with vehicle or curcumin (200 mg/kg). Mice were treated for seven consecutive days, repeated weekly for 4 weeks. Data are presented as mean tumor volume ± standard error of the mean (SEM) (n = 6; P < 0.05). A representative experiment is shown. (**D–E**) Tumors were removed from control untreated or curcumin-treated and/not KU-55933-treated mice, followed by immunofluorescence analysis with antibodies against Caspase-3 and Ki-67 and CD31. Micrographs are representative of tumor sections from two different mice in each group. (**F–H**) We further investigated the effect of curcumin on MMP-2 expression and VEGF production in curcumin-treated and/not KU**-**55933-treated mice. Whole-tissue lysates were subjected to western-blot analysis for determining levels of total MMP-2 and VEGF proteins. Pretreatment of cancer cells with either curcumin alone, or combination of curcumin and KU55933 significantly inhibited MMP-2 and VEGF protein expression. Quantitative reverse transcription–PCR of MMP-2 and VEGF in cancer cells demonstrates that MMP-2 and VEGF mRNA was down-regulated expression. GAPDH was used as an internal loading control. The data are expressed as mean ± SD of three independent experiments, and significant differences from the control are indicated by **p < 0.01.

To further validate the interaction between curcumin and SIRT1 *in vivo*, xenograft mouse model method was developed to evaluate potential anti-tumor activity of curcumin. Having shown that curcumin induced apoptosis in FaDu and Cal27 cells *in vitro*, we next examined the *in vivo* efficacy of curcumin treatment using FaDu cells xenograft mouse model. A significant reduction in tumor growth was observed in mice receiving curcumin treatment relative to untreated mice (Fig. 7A–C). Examination of the xenografted tumor sections showed that curcumin increased the number of cleaved-caspase-3 positive cells. Furthermore, a significant decrease in proliferation marker Ki-67-positive cells was also noted in tumor sections from curcumin-treated mice relative to control mice. We investigate the potential capacity of curcumin to inhibit angiogenesis though measuring CD31 expression levels in xenografted cells in nude mice (Fig. 7D–E). Inhibition of the SIRT1 pathway resulted in down-regulation and suppression of MMP-2 and VEGF protein. Quantitative reverse transcription–PCR of MMP-2 and VEGF in cancer cells demonstrates that MMP-2 and VEGF mRNA was down-regulated expression (Fig. 7F–H). We found no toxicity in the FaDu cells xenograft model even upon treatment of mice with curcumin at 200 mg/kg doses. These data demonstrate potent anti-tumor activity of curcumin *in vivo*.

### Discussion

Curcumin can be used to inhibit the growth of cancer cells due to their ability to affect the activity of multiple targets involved in carcinogenesis^31^,^32^,^33^,^34^. Epidemiological studies have shown the association between the consumption of fruits and vegetables and the prevention of human diseases, including cancer. Some study found that the treatment of HNSCC cells with combinations of curcumin and transresveratrol can be more effective in inhibiting *in vivo* and *in vitro* cancer cell growth than the treatment with curcumin alone^35^,^36^.

The sirtuins (SIRTs), also known as silent information regulator-2 (Sir2) proteins, consists of nicotinamide adenine dinucleotide (NAD^+^)-dependent deacetylases (class III) that are involved in various cellular processes from aging to cancer^37^. The anticancer potential of curcumin stems from its ability to suppress proliferation of a wide variety of tumor cells, down-regulate transcription factors NF-κB, AP-1 and Egr-1; down-regulate the expression of COX2, LOX, NOS, MMP-9, uPA, TNF, chemokines, cell surface adhesion molecules and cyclin D1; down-regulate growth factor receptors (such as EGFR and HER2); and inhibit the activity of c-Jun N-terminal kinase, protein tyrosine kinases and protein serine/threonine kinases^38^,^39^. Previous studies have demonstrated the anti-tumour activity of SIRT1 activators^11^,^24^. There is mounting evidence that curcumin can suppress tumor initiation, promotion and metastasis^38^. Our data showed that curcumin markedly increases the SIRT1 deacetylating enzymatic activity in HNSCC cells; conversely, pre-treatment of cells with nicotinamide blocked curcumin-triggered activity. Although some controversy exists concerning the specificity of curcumin toward SIRT1^25^, the notion that SIRT1 is a target of curcumin is supported by recent data showing that it activates SIRT1 via a allosteric mechanism^38^. Taken together, these results illustrate the involvement of multiple SIRT1-dependent mechanisms in the biological actions of curcumin.

In the present study, we provide evidence that curcumin inhibits the proliferation and induces apoptosis of HNSCC cell lines. Importantly, our data demonstrate anticancer activity of curcumin in HNSCC cell lines, as well as representing distinct cytogenetic profiles^31^. Our study showed that curcumin inhibited migration and tube formation of carcinoma cells. Is it due to the induction of apoptosis? If there is an important apoptotic effect of curcumin in carcinoma cells, it is obvious that their migratory ability have been impaired. In addition, the ATM abnormalities or p53 status in HNSCC cells may contribute to curcumin anticancer activity, and this issue remains to be defined^22^,^40^. Overall, our present study showed that curcumin-induced apoptosis in FaDu and Cal27 cells occurs via both mitochondria-independent and mitochondria-dependent apoptotic signal pathway.

Moreover, SIRT1 plays an important role in DNA damage response and DNA repair activity. The DNA damage response is mediated via activation of ATM, a serine/threonine protein kinase that is recruited and activated by DNA double-strand breaks^22^. ATM phosphorylates several key proteins that activate DNA damage checkpoint, thereby triggering cell cycle arrest, DNA repair and apoptosis. Our data above demonstrating that anticancer activity of curcumin is associated with activation of the caspase cascade and apoptotic signal involving the ATM/CHK2 pathway^41^. Blockade of ATM abrogated curcumin-induced HNSCC cell death. Overall, our current study suggests that the anticancer activity of curcumin involves the ATM-CHK2/caspase 8/9 pathway^42^. The role of SIRT1 in this ATM/CHK2 pathway has further demonstrated the role of SIRT1 in the regulation of DNA damage response. Although ATM-CHK2 signal plays a key role in mediating apoptosis triggered by curcumin, the possibility that other signal pathways are also activated by curcumin cannot be excluded. The NF-κB pathway is often aberrantly activated during the development and progression of HNSCC^43^. Activated NF-κB binds DNA as a heterodimeric complex composed of members of the Rel/NF-κB family of polypeptides. Because of its intimate involvement in host defense against disease, this transcription factor is an important target for therapeutic intervention. In the present report we demonstrate that curcumin is a potent inhibitor of NF-κB activation. IκB-α is a key molecular target regulating NF-κB activation^39^,^44^. When NF-κB signal was blocked by curcumin in this study, curcumin significantly inhibited FaDu and Cal27 cells growth in a dose-dependent fashion. This result strongly suggests that NF-κB mediates protection against curcumin induced apoptosis in HNSCC cells.

Finally, taking into account that a limited number of animal studies are available on the anti-cancer effects of curcumin, we evaluated the *in vivo* effects of curcumin treatment in hampering the growth of transplanted FaDu cells in athymic nude (nu/nu) mice. A relative growth inhibitory effect of curcumin was observed in a human FaDu cell xenograft mouse model. In agreement with other animal model studies using SIRT1-activating agents^45^, no toxicity was observed during treatment of mice with curcumin at 200 mg/kg doses. The remarkable anticancer activity of curcumin *in vivo* was confirmed by immunofluorescence analysis of tumors harvested from control and curcumin-treated mice using molecular markers of apoptosis and proliferation (caspase-3 and Ki-67 respectively). Inhibition of the SIRT1 pathway resulted in down-regulation and suppression of MMP-2 and VEGF protein. These results suggested that the curcumin mediated *in vivo* effects may occur through a molecular mechanism dependent of SIRT1 activation and confirmed our *in vitro* data suggesting the potent anticancer activity of curcumin.

In summary, our studies demonstrate potent *in vitro* and *in vivo* anticancer activity of curcumin in a SIRT1-dependent fashion. This work highlights the importance of examining the therapeutic value of small molecule activators of SIRT1 in HNSCC.

## Additional Information

**How to cite this article**: Hu, A. *et al.* Curcumin as therapeutics for the treatment of head and neck squamous cell carcinoma by activating SIRT1. *Sci. Rep.* **5**, 13429; doi: 10.1038/srep13429 (2015).
